# OTUB1 de-ubiquitinating enzyme promotes prostate cancer cell invasion *in vitro* and tumorigenesis *in vivo*

**DOI:** 10.1186/s12943-014-0280-2

**Published:** 2015-01-27

**Authors:** Diego Iglesias-Gato, Yin-Choy Chuan, Ning Jiang, Charlotte Svensson, Jing Bao, Indranil Paul, Lars Egevad, Benedikt M Kessler, Pernilla Wikström, Yuanjie Niu, Amilcar Flores-Morales

**Affiliations:** The Novo Nordisk Foundation Center for Protein Research, Faculty of Health Sciences, University of Copenhagen, 2200 Copenhagen, Denmark; Tianjin Institute of Urology, Tianjin Medical University, 300211 Tianjin, China; Section of Urology, Department of Surgical Science Karolinska Institutet, 17176 Stockholm, Sweden; Target Discovery Institute, Nuffield Department of Clinical Medicine, University of Oxford, OX3 7BN Oxford, UK; Department of Medical Biosciences, Pathology, Umeå University, 90185 Umeå, Sweden

**Keywords:** Otubain 1, Deubiquitinase, Ubiquitin, Prostate cancer, RhoA, Androgen

## Abstract

**Background:**

Ubiquitination is a highly dynamic and reversible process with a central role in cell homeostasis. Deregulation of several deubiquitinating enzymes has been linked to tumor development but their specific role in prostate cancer progression remains unexplored.

**Methods:**

RNAi screening was used to investigate the role of the ovarian tumor proteases (OTU) family of deubiquitinating enzymes on the proliferation and invasion capacity of prostate cancer cells. RhoA activity was measured in relation with OTUB1 effects on prostate cancer cell invasion. Tumor xenograft mouse model with stable OTUB1 knockdown was used to investigate OTUB1 influence in tumor growth.

**Results:**

Our RNAi screening identified OTUB1 as an important regulator of prostate cancer cell invasion through the modulation of RhoA activation. The effect of OTUB1 on RhoA activation is important for androgen-induced repression of p53 expression in prostate cancer cells. In localized prostate cancer tumors OTUB1 was found overexpressed as compared to normal prostatic epithelial cells. Prostate cancer xenografts expressing reduced levels of OTUB1 exhibit reduced tumor growth and reduced metastatic dissemination *in vivo.*

**Conclusions:**

OTUB1 mediates prostate cancer cell invasion through RhoA activation and promotes tumorigenesis *in vivo*. Our results suggest that drugs targeting the catalytic activity of OTUB1 could potentially be used as therapeutics for metastatic prostate cancer.

**Electronic supplementary material:**

The online version of this article (doi:10.1186/s12943-014-0280-2) contains supplementary material, which is available to authorized users.

## Background

Prostate cancer (PCa) is one of the leading causes of cancer related death in the western world [1,2]. Mortality in PCa is due to our inability to provide efficient therapies for the management of the metastatic disease associated with the development of castration-resistant prostate cancer (CRPC). The mechanisms governing metastatic progression in PCa are poorly understood, although recurrent genetic changes leading to PTEN and p53 inactivation and to overexpression of c-myc are often observed in advanced tumors [3,4]. Despite being resistant to androgen ablation therapy, most CRPC tumors retain the expression of functional androgen receptor (AR), which contributes to the growth of these tumors through multiple mechanisms [5-10]. The AR can cooperate with oncogenes to promote tumorigenesis, e.g., we have recently shown that AR activation leads to increased PCa cell invasion through inhibition of c-myc proteasomal degradation [11].

Protein ubiquitination is a highly regulated process that controls multiple physiologically and pathologically relevant mechanisms involved in tumor development. The degree of ubiquitination of specific proteins is controlled by the concerted actions of E3 ubiquitin ligases, de-ubiquitinating enzymes (DUBs) and the proteasome [12,13]. DUBs specifically cleave the isopeptide bonds of polyubiquitin moieties. Therefore, DUBs are an important component in controlling the extent and nature of ubiquitin chains built on specific proteins, in addition to their function in the recycling of ubiquitinated precursors [13]. The OTU domain containing DUBs (OTUDs) are cysteine-dependent proteases with poorly characterized cellular functions. An understanding of OTUDs enzymes role in carcinogenesis is just starting to emerge. It has been shown that OTUB1 and OTUB2 can regulate DNA damage response and OTUB1 has been found elevated in colon carcinomas [14-16]. The A20 OTUD regulates NFκB activity and plays an important role in lymphomas [17], while deregulation of OTUD1 has been shown in thyroid carcinomas [18]. The cellular OTUDs targets, responsible for their effects on tumorigenesis are generally unknown. There is little evidence that high affinity protein-protein interactions beyond those involving polyubiquitin side chains contribute to the specific recognition of substrates by OTUDs, making target identification difficult. On the other hand, it has been shown that different OTUDs exhibit certain specificity towards different types of ubiquitin linkages [19]. For example, OTUB1 preferentially processes polyubiquitin chains linked by Lys48 while OTUB2 favors Lys63 bonds. TRABID cleaves Lys29 and Lys33, Cezanne shows more activity towards Lys11 linkages and Otulin cleaves linear ubiquitin [19,20]. In addition to its canonical activity as polyubiquitin proteases, some OTUDs (e.g OTUB1) can directly bind and inhibit E2 ubiquitin conjugating enzymes independently of their enzymatic activity [15,21].

Here we investigated whether members of the ovarian tumor proteases (OTU) family of DUBs influence the proliferation and invasion capacity of PCa cells. We found that OTUB1 plays a critical role not only in the AR-dependent but also AR-independent cell invasion of prostate cancer cells *in vitro* and *in vivo* through the modulation of RhoA activity. Besides, the analysis of prostate cancer clinical samples shows that OTUB1 is overexpressed in localized tumor as compared to normal prostate epithelial cells.

## Results

### siRNA screening identifies OTUB1 as a novel regulator of prostate cancer cells invasion

**Figure 1:**
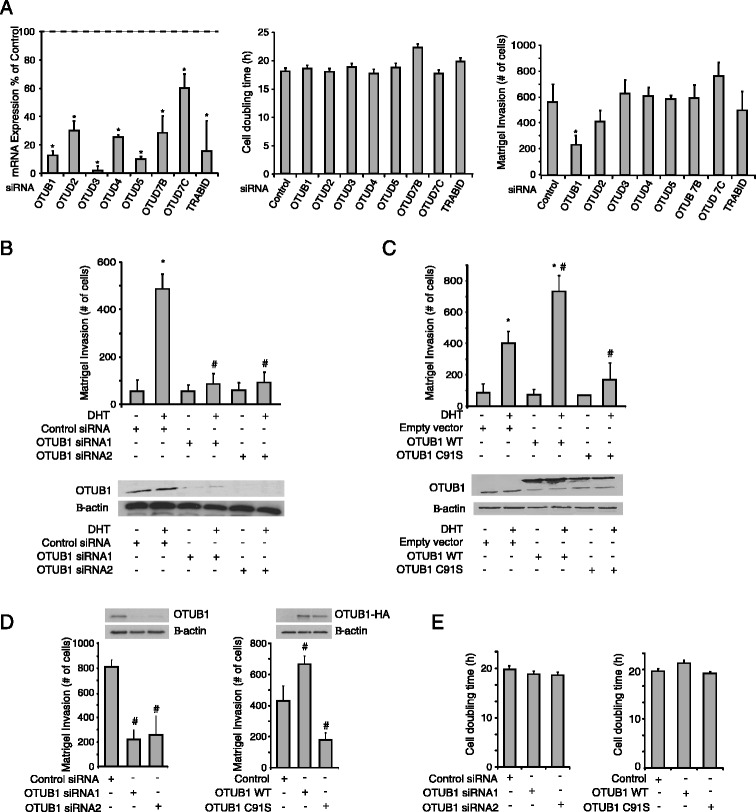
**Functions of the OTU-domain containing proteins in prostate cancer progression. (A)** LNCaP-FGC cells transfected with siRNAs targeting different OTU family members were assayed for: (left panel) the knockdown efficiency of the siRNAs measured by quantitative real-time PCR -results are shown as percentage expression of each gene relative to siRNA control transfected cells; (middle panel) cell proliferation and (right panel) matrigel cell invasion. **(B)** Matrigel invasion assay (upper panel) and western blot analysis of OTUB1 expression (lower panel) in LNCaP-FGC cells transfected with two different siRNAs targeting OTUB1 or a control siRNA, treated with DHT (10 nM). **(C)** Same as in **(B)** using LNCaP-FGC cells transfected with either an empty vector or expression plasmids for wild type OTUB1 (OTUB1-WT) or the protease inactive C91S (OTUB1-C91S) variant. **(D)** Measurements of matrigel invasion and **(E)** cell proliferation of PC3 cells transfected with either siRNAs targeting OTUB1 or expression vectors bearing OTUB1-WT or the C91S variant, as indicated. In all panels, each column represents the average ± SD of at least four independent replicates and Student´s t test was used for statistical analysis. In **(A)**, *indicates a significant reduction (*p* < 0.05) of mRNA levels and invasive capacity between LNCaP-FGC cells transfected with control siRNA compared with the different OTUs siRNAs. In **(B)** and **(C)**, *indicates a statistically significant (*p* < 0.05) change after DHT treatment in cells transfected with the same siRNA or plasmid. ^#^indicates a significant (*p* < 0.05) change between cells transfected with siOTUB1, OTUB1-WT or OTUB1-C91S and control transfected cells upon DHT stimulation. In **(D)**, ^#^indicates a significant (*p* < 0.05) change between cells transfected with siOTUB1, OTUB1-WT or OTUB1-C91S versus control transfected cells.

We wanted to investigate the potential roles of OTU-domain containing proteins with cysteine protease function (OTUD) in prostate cancer cells tumorigenesis. Therefore, we performed a small interfering RNA (siRNA)-based screening against a panel of OTU family members -OTUB1, OTUB2, OTUD3, OTUD4, OTUD5, OTUD7B and OTUD7C and TRABID- to measure their influence in the proliferation and invasion capacity of LNCaP-FGC cells. The efficiency of the knockdown was assessed by measuring the reduction of mRNA levels of each gene compared to scrambled siRNA transfected controls. After transfecting with the siRNA pools, at least 70% reduction was observed for all OTUD mRNAs but for OTUD7C mRNA (40%) (Figure 1A, left panel). Transient transfection of the aforementioned siRNAs into LNCaP-FGC cells didn’t result in a significant alteration of cell proliferation *in vitro* (Figure 1A, middle panel). LNCaP-FGC cells show a low capacity to invade through matrigel *in vitro*, which can be significantly stimulated by dihydrotestosterone (DHT) treatment [11]. Therefore, we tested the effects of the OTUD family targeting siRNAs in DHT-induced invasion capacity of LNCaP-FGC cells. We found that of all the siRNAs tested, only the inhibition of OTUB1 expression was able to significantly affect cell invasion of LNCaP-FGC cells in presence of DHT (Figure 1A, right panel).

To confirm this observation, two additional individual siRNAs targeting OTUB1 were used. As shown in Figure 1B, siRNAs targeting OTUB1 but not control siRNA reduced OTUB1 protein expression levels while inhibiting the invasive capacity of LNCaP-FGC cells upon DHT stimulation. Moreover, overexpression of the protease inactive OTUB1-C91S mutant [22] also resulted in diminished invasiveness (Figure 1C), supporting the regulatory role of OTUB1 in prostate cancer cell invasion. In order to validate these observations as general phenomena in prostate cancer cells, we analyzed the effects of OTUB1 in the androgen-insensitive PCa cell line, PC3. We confirmed that both OTUB1 knockdown and overexpression of the catalytically inactive mutant results in diminished invasive activity while having no effect on cell proliferation (Figure 1D, E). Similar effects were observed using 22Rv1 prostate cancer cells (Additional file 1: Figure S1) supporting the role of OTUB1 in the regulation of prostate cancer cells invasion.

### Elevated OTUB1 protein levels in prostate cancer

**Figure 2:**
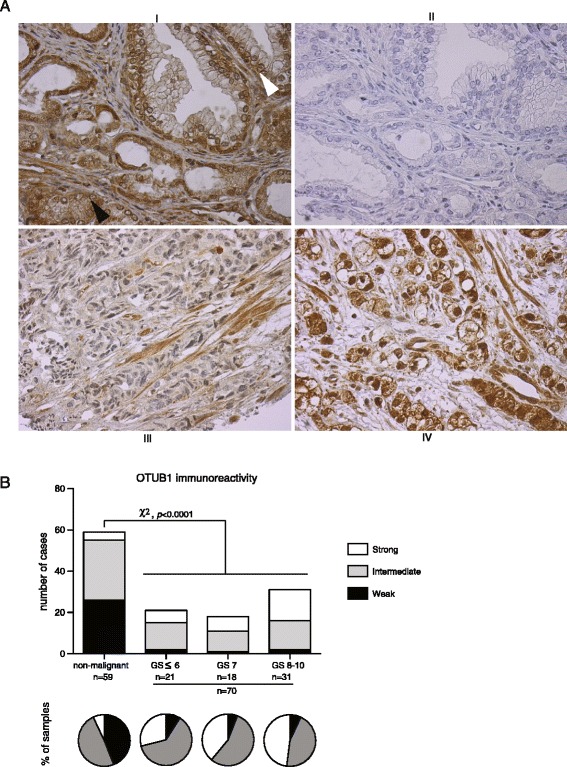
**OTUB1 expression in prostate cancer. (A)** Immunohistochemical analysis of OTUB1 expression in prostate cancer. I, prostate sample showing a gland with normal architecture (white arrow head) and a tumor area with a Gleason score of 6 (black arrow head). In the normal gland high OTUB1 expression is observed in basal epithelial cells and in adjacent stromal cells, and increased expression is observed in tumor epithelial cells compared to normal luminal epithelium. II, negative control staining for A. III, example of a prostate tumor with Gleason score of 8 exhibiting moderate staining of epithelial tumor cells. IV, a tumor with Gleason score of 9 showing strong staining of tumor cells. **(B)** Distribution of OTUB1 expression across prostate tissue samples with different Gleason grades are shown as bar graphs while relative numbers are shown as pie charts. Chi-square *p* value comparing OTUB1 expression in malignant versus non-malignant prostate tissues and OTUB1 expression across the different histological Gleason score grades are shown. OTUB1 IR is independent of Gleason score; Chi-square *p* value across Gleason score grades is 0.7.

We next analyzed OTUB1 expression in prostate cancer by immunohistochemistry using tissue microarrays (TMAs) (Figure 2). OTUB1 immunoreactivity (IR) was scored as weak, intermediate or strong (Figure 2A). Analysis of non-malignant tissue (n = 59) showed strong staining of basal epithelial and stromal cells, while the staining of luminal epithelial cells was mainly weak (44%) or intermediate (49%) (Figure 2B). On the other hand, 40% of the tumors (n = 70) exhibited strong OTUB1 IR in luminal cells while only 7% of the cases showed weak IR (Figure 2B). This significant increase (Chi-square test, *p* < 0.0001) in OTUB1 IR in the malignant tissue was independent from the histological grade, Gleason score (Figure 2B). Furthermore, some prostate cancer samples exhibited strong nuclear staining. The demonstration that OTUB1 is overexpressed in PCa suggests a role for OTUB1 in tumorigenesis and invites additional exploration of its mechanisms of action.

### OTUB1 positively regulates androgen signaling in LNCaP-FGC cells

**Figure 3:**
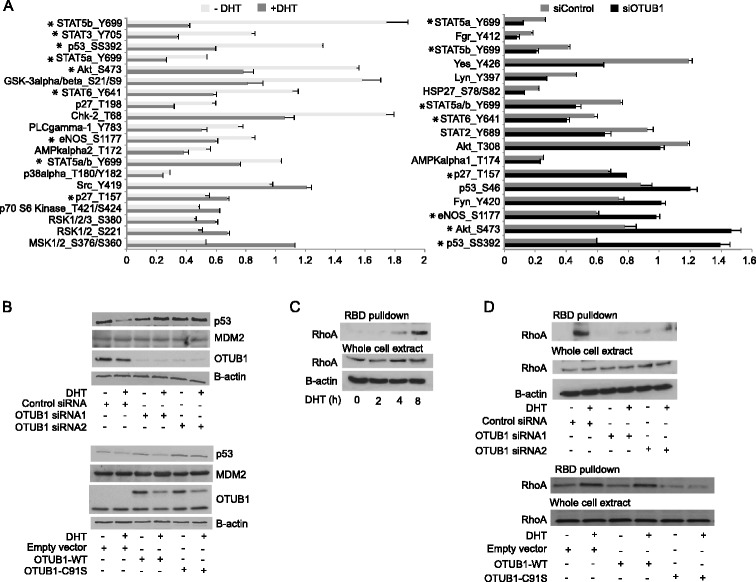
**Androgens and OTUB1 regulate RhoA activity and p53 protein levels in PCa cells. (A)** Phospho-protein array analysis of changes in protein phosphorylation in LNCaP-FGC cells transfected with control siRNA or an OTUB1 targeting siRNA, treated or not with DHT. Left panel shows the effects of DHT on siRNA control transfected cells and in the right panel the effects of different siRNAs on DHT treated cells are compared. Measurements were performed in duplicates. Students´ t test was applied to evaluate the statistical significance of the phosphorylation changes consequence of DHT (left panel) and OTUB1 depletion (right panel). *p* < 0.05 was considered as significant (*). **(B)** Analysis of p53, MDM2, OTUB1 and Beta-actin expression by western blot in extracts obtained from LNCaP-FGC cells transfected with siRNA targeting OTUB1 or siRNA control (upper panel) and OTUB1-WT, OTUB1-C91S variant or empty vector (lower panel) treated with or without DHT. **(C)** RhoA activity assay on cell extracts purified from LNCaP-FGC cells stimulated with DHT at different time points as indicated. **(D)** RhoA activity assay as in **(C)** using extracts from LNCaP-FGC cells transfected and treated as indicated.

We used a phospho-antibody array to explore possible mechanisms by which OTUB1 regulates cell invasion in response to DHT treatment. We analyzed changes in the phosphorylation pattern of 46 signaling proteins in extracts from LNCaP-FGC cells transfected with OTUB1 or control siRNA and treated with or without DHT. Because DHT positively regulates cell invasion in LNCaP-FGC cells [3], we reasoned that pathways regulated by OTUB1 knockdown that exhibit opposite regulation by DHT treatment might be of relevance for the regulation of cell invasion. As shown in Figure 3A, we found that upon DHT treatment cells transfected with control siRNA showed a significant induction of MSK phosphorylation (S376/S360), and a more modest induction of Src (Y419), RSK1/2 (S221), RSK1/2/3 (S380), p27 (T157) and p70-S6 Kinase (T421/S424) phosphorylation. On the other hand, we detected a significant reduction in the phosphorylation levels of STAT5b (Y699), STAT6 (Y641), STAT3 (Y705), PLCγ1 (Y783), p53 (S392), p27 (T198), GSK3α/β (S21/S9), eNOS (S1177), Chk2 (T172) and AKT1 (Ser473). Interestingly, OTUB1 knockdown in the presence of DHT opposed the effects of androgens resulting in a significant induction of p53 (S392), AKT (Ser473) and eNOS (S1177) phosphorylation level (Figure 3A).

The influence of OTUB1 on androgen signaling was further confirmed by measuring changes in the total proteome upon OTUB1 knockdown. LNCaP-FGC cells were metabolically labeled in 3 isotopic configurations: “heavy”, “medium” and “light”, using the stable isotope labeling by amino acids in cell culture (SILAC) methodology (Methods section and [23]). “Heavy” cells harbored proteins containing Arginine 10 (Arg10) and Lysine 8 (Lys8) isotopes; “medium” cells carried Arg6/Lys4 isotopes and “light” cells had Arg0/Lys0 as the only isotopic variants of these amino acids. “Heavy” and “medium” cells were then transfected with siRNAs targeting OTUB1 while “light” cells were transfected with control siRNA. Protein extracts of the three cell lines were combined in 1:1:1 ratio and digested with trypsin. Resulting peptides were analyzed by mass spectrometry. Tryptic peptides are expected to contain single Arg or Lys. Therefore, peptides originated from “heavy”, “medium” and “light” cells will differ in mass accordingly to the isotopic labeling. Intensity comparison of isotopic peptides will accurately reflect differences in peptide abundance [24]. Interestingly, we observed an overrepresentation of androgen-regulated proteins (9/34) among those with reduced expression after OTUB1 knockdown (e.g. Prostatic acid phosphatase, ACPP [25]; prostate specific antigen, KLK3; MALT1; NDRG1 [26]) (Table 1). These results supported the novel function of OTUB1 as positive mediator of androgen signaling.

**Table 1:** **Proteins regulated upon OTUB1 knockdown in LNCaP-FGC cells**

**Gene name**	**Uniprot**	**Log2(siOTUB1_1/siControl)**	**Log2(siOTUB1_2/siControl)**	**Mean (SD)**
**ACPP**	P15309	−7.23	−3.08	−5.15 (2.94)
**ACTA2**	P62736	−3.52	−5.65	−4.58 (1.51)
**SHROOM3**	Q8TF72	−3.75	−3.24	−3.49 (0.36)
**KLK3**	P07288	−3.52	−2.72	−3.12 (0.57)
TAGLN	Q01995	−2.44	−3.36	−2.9 (0.65)
DES	P17661	−3.69	−1.95	−2.82 (1.23)
KPRP	Q5T749	−2.33	−2.75	−2.54 (0.3)
ST14	Q9Y5Y6	−1.03	−3.95	−2.49 (2.07)
DCD	P81605	−2.60	−2.34	−2.47 (0.19)
CASP14	P31944	−3.23	−1.44	−2.34 (1.27)
STAM	Q92783	−1.36	−2.18	−1.77 (0.58)
ACTC1	P68032	−1.05	−2.36	−1.7 (0.93)
ANXA2	P07355	−1.17	−2.17	−1.67 (0.71)
HSPB1	P04792	−1.20	−1.91	−1.56 (0.5)
DTNB	O60941	−1.57	−1.55	−1.56 (0.02)
SNX2	O60749	−0.82	−1.84	−1.33 (0.72)
BLMH	Q13867	−1.44	−1.09	−1.27 (0.25)
**MICAL1**	Q8TDZ2	−1.02	−1.43	−1.23 (0.29)
WDR7	Q9Y4E6	−1.17	−1.26	−1.21 (0.06)
**MALT1**	Q9UDY8	−1.28	−0.99	−1.13 (0.2)
RAB8A	P61006	−0.75	−1.23	−0.99 (0.34)
NOC2L	Q9Y3T9	−1.04	−0.89	−0.96 (0.1)
ARHGAP35	Q9NRY4	−1.19	−0.73	−0.96 (0.32)
UBE2K	P61086	−1.09	−0.81	−0.95 (0.2)
USP39	Q53GS9	−1.19	−0.69	−0.94 (0.36)
**HMGCS2**	P54868	−0.72	−1.12	−0.92 (0.28)
QSOX1	O00391	−0.92	−0.89	−0.9 (0.02)
TOR1AIP1	Q5JTV8	−0.75	−0.91	−0.83 (0.11)
**ADAM9**	Q13443	−0.86	−0.73	−0.79 (0.09)
LAMC1	P11047	−0.77	−0.80	−0.79 (0.02)
NUP88	Q99567	−0.71	−0.86	−0.79 (0.1)
YWHAB	P31946	−0.68	−0.73	−0.71 (0.03)
**NDRG1**	Q92597	−0.65	−0.75	−0.7 (0.07)
FAM160B1	Q5W0V3	−0.66	−0.66	−0.66 (0)
DBR1	Q9UK59	0.65	0.59	0.62 (0.05)
SLC4A2	P04920	0.89	0.75	0.82 (0.1)
POLA1	P09884	0.69	1.14	0.91 (0.32)
DDX42	Q86XP3	0.92	1.13	1.02 (0.15)
TTN	Q8WZ42	1.50	1.22	1.36 (0.2)
PTGFRN	Q9P2B2	2.04	1.66	1.85 (0.27)

Proteins showing changes in expression after OTUB1 knockdown using two different siRNA, compared to control siRNA transfected LNCaP-FGC cells. Androgen regulated proteins are highlighted. Fold changes are shown as log2 of the ratio between the intensity of the proteins in the siRNA transfected and control siRNA transfected cells. Mean values and Standard deviation (SD) for both experiments are shown on the right column.

### OTUB1 regulates RhoA activity and p53 levels in LNCaP-FGC cells

In a previous study we described the capacity of OTUB1 to modulate bacterial uptake through the modulation of RhoA activity [27]. Reduced levels of p53 have been associated with increased fibroblast cell motility through the activation of the RhoA small GTPase [28]. Our findings that p53 (S392) phosphorylation levels are reduced in LNCaP-FGC cells by DHT stimulation and stimulated by OTUB1 knockdown, suggest that the p53/RhoA pathways may also be involved in androgen regulation of cell invasion. In order to test this hypothesis, we first analyzed p53 protein levels in LNCaP-FGC cells expressing OTUB1 siRNAs or an OTUB1 catalytically inactive variant (OTUB1-C91S). As shown in Figure 3B, p53 protein levels decreased in DHT stimulated cells, while these effects are blocked by OTUB1 siRNA or forced overexpression of OTUB1-C91S mutant. Interestingly, these changes in p53 abundance are not accompanied with alteration in the levels of the p53-E3-ubiquitin ligase MDM2. It is also important to notice that OTUB1 depletion in the absence of androgen stimulation does not further increase p53 expression (Figure 3B). These results indicate that OTUB1 activity modulate androgen actions on the regulation of p53 expression in PCa cells.

Next, we studied the influence of androgen treatment on RhoA activity in LNCaP-FGC cells through the quantitation of the GTP-bound RhoA fraction (Figure 3C). The experiments show that DHT treatment increased the level of active RhoA but not the total RhoA levels, while no effects were observed for the GTPases Rac and Cdc42 (data not shown). On the other hand, a significant reduction of active but not total RhoA levels was observed in cells transfected with two different siRNAs targeting OTUB1 or with the inactive OTUB1-C91S variant (Figure 3D), supporting a role for OTUB1 in the regulation of RhoA activation by androgens.

### RhoA mediates the effects of OTUB1 on cell invasion and the regulation of p53 levels

**Figure 4:**
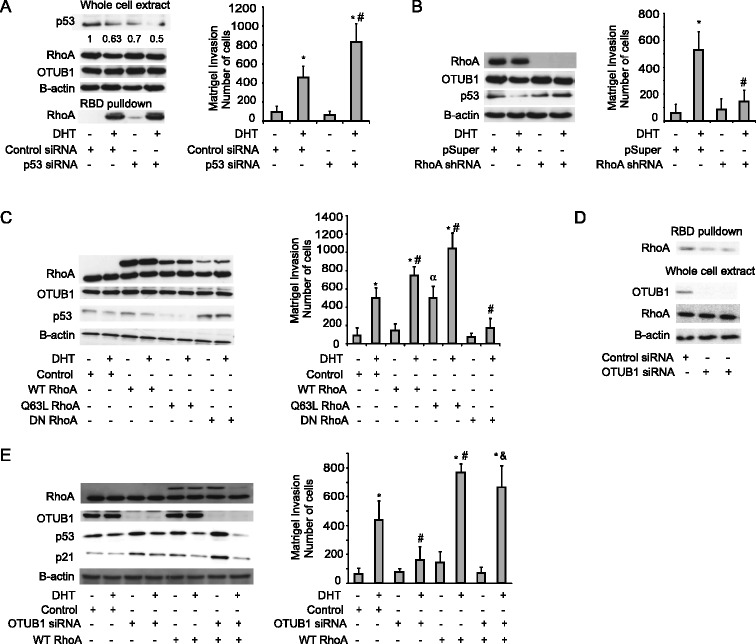
**RhoA activity mediates the effects of OTUB1 on p53 expression and matrigel invasion in PCa cells. (A)** Western blot analysis for the indicated proteins and RhoA activity assay (left panel) of LNCaP-FGC cells transfected with p53 siRNA or control siRNA and treated with or without DHT. Matrigel invasion assay for the same cells is shown on the right. Quantification of p53 expression relative to beta-Actin levels is indicated. p53 levels in untreated-control transfected cells were set as 1. Western blot and matrigel invasion assays as in **(A)** were performed using cells transfected with RhoA shRNA or control plasmid **(B)**, wild type (WT), constitutively active (Q63L) or dominant negative (DN) alleles of RhoA **(C)** and the combination of WT-RhoA and siRNA against OTUB1 **(E)** in the presence of DHT or control vehicle. **(D)** RhoA activity assay as in **(A)** on PC3 cells transfected with siRNAs targeting OTUB1 or control siRNA. In invasion assays, each column represents the average ± SD of at least four independence replicates. *indicates statistically significant (*p* < 0.05) changes after DHT treatment in cells transfected with the same siRNA or plasmid. ^#^indicates significant (*p* < 0.05) differences between control transfected cells versus cells tranfected with other siRNAs or plasmids in the presence of DHT. & indicates significant (*p* < 0.05) differences between cells transfected with siOTUB1 and cells transfected with siOTUB1 and WT-RhoA in the presence of DHT. ^α^indicates significant (*p* < 0.05) differences between cells transfected with Q63L-RhoA and control cells in the absence of DHT.

Having identified p53 and active RhoA as OTUB1 regulated proteins in LNCaP-FGC cells, we next investigated the role of these proteins in androgen induced cell invasion. First, we used siRNAs to test the effects of reduced levels of p53 on the DHT regulation of RhoA activity and matrigel invasion. As shown in Figure 4A, transfection with p53 siRNA causes a reduction in expression levels of p53 comparable to that observed after DHT treatment in cells transfected with control siRNA (~30% reduction). Downregulation of p53 either by siRNA or by DHT treatment does not alter total levels of OTUB1 or RhoA. On the other hand, p53 knockdown leads to a small increase in the amount of GTP bound RhoA in the absence of androgen treatment, although the levels were significantly lower than those observed upon DHT treatment and insufficient to significantly promote cell invasion in the absence of DHT stimulation (Figure 4A, right panel). Treatment with DHT of cells transfected with p53 targeting siRNA leads to further reduction in the levels of p53 (50% reduction) and enhanced cell invasion. The knockdown of p53 did not cause a significant increase on RhoA activation by DHT in comparison with control cells treated with the hormone.

Having failed to observe major effects of p53 downregulation on RhoA activity levels, we next analyzed whether RhoA activity influences p53 actions in prostate cancer cells. We measured p53 levels in cells where RhoA activity was inhibited either by expression of shRNA targeting RhoA or through the expression of a dominant negative form of RhoA (DN-RhoA). As shown in Figure 4B, reduced expression of RhoA in DHT treated cells leads to increased p53 protein levels as compared with DHT treated control cells. This effect is concomitant to a reduction in cell invasion, confirming a role of RhoA in the regulation of both p53 levels and cell invasiveness in prostate cancer cells. Similar results were obtained when analyzing cells expressing DN-RhoA (Figure 4C). These cells exhibit elevated p53 protein levels both in the absence and presence of DHT and show reduced cell invasiveness upon DHT stimulation, despite the fact that significant amounts of endogenous wild type levels of RhoA are expressed in these cells. Conversely, the overexpression of RhoA constitutive active form (Q63L-RhoA) resulted in reduced levels of p53 and enhanced cell invasiveness even in cells deprived of DHT. From these experiments we can conclude that RhoA activation is essential for DHT induction of cell invasion and also mediates the effects of DHT on the regulation of p53 protein levels.

Our results also suggest that OTUB1 effects on RhoA activation are independent of changes in p53 levels. We tested this hypothesis further by analyzing PC3 prostate cancer cells, whose invasive capacity is modulated by OTUB1 (Figure 1C), but lacks expression of p53 and AR. We showed that cells transfected with two independent OTUB1 targeting siRNAs exhibit significantly lower GTP bounded RhoA as compared to cells transfected with control siRNA (Figure 4D), confirming that OTUB1 regulation of RhoA also occurs independently of p53 or the AR.

In the next experiments, we tested whether the OTUB1 effects on cell invasion are driven through the regulation of RhoA activity. We analyzed whether overexpression of RhoA can overcome the inhibitory effects of OTUB1 siRNA-based knockdown on cell invasion. As shown in Figure 4E, overexpression of WT-RhoA slightly, although significantly, induces the invasive capacity of LNCaP-FGC cells upon DHT stimulation. Importantly, WT-RhoA rescues the inhibition of the invasive activity observed after OTUB1 knockdown in DHT treated cells. In parallel, RhoA overexpression counteracts the effect of OTUB1 knockdown on the levels of p53 and its downstream target p21. These findings confirm that RhoA is an important mediator of OTUB1 effects on cell invasion and p53 expression.

### OTUB1 regulates anchorage independent growth *in vitro* and tumor development *in vivo*

**Figure 5:**
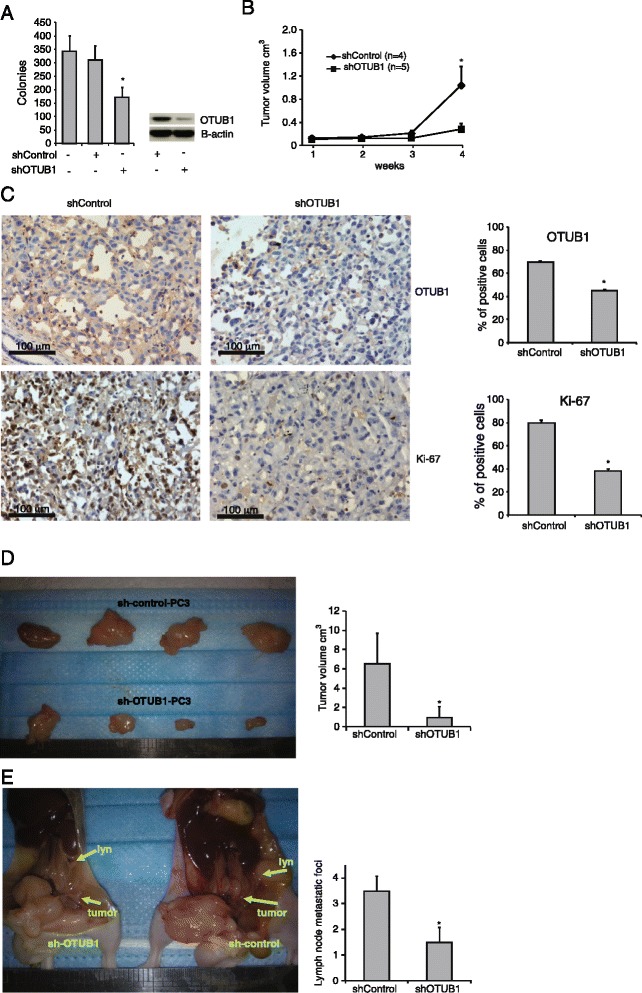
**PC3 cells xenografts expressing shOTUB1 show decreased tumorigenesis and metastatic activity. (A)** PC3 cells expressing a short harpin cDNA targeting OTUB1 or a scramble control were used to measure anchorage independent growth in soft agar. **(B)** PC3-shOTUB1 or PC3-shControl cells were implanted subcutaneously (n = 4 mice per group) and tumor growth was measured weekly. **(C)** Immunohistochemical analysis of the expression of OTUB1 and Ki-67 in the xenograft tumors. Three random areas per mouse and tumor were counted for the expression of both proteins and the results are shown as percentage of positive cells. **(D)** PC3-shOTUB1 or PC3-shControl cells were implanted into the prostate of nude mice (n = 4). After 8 weeks, orthotopic tumors were surgically removed and their volumes analyzed. **(E)** Number of lymph node metastatic foci per mouse. *indicates statistically significant (*p* < 0.05) changes between shOTUB1 cells and shControl cells.

In order to test the possible function of OTUB1 in the regulation of tumor growth *in vivo*, we generated PC3 cells that stably express a short hairpin (sh) RNA targeting OTUB1 expression or a scramble control shRNA. First, we tested these cells for their anchorage independent growth capacity. Inhibition of OTUB1 expression in PC3 cells significantly reduced the number of colonies formed in soft agar as compared to control cells (Figure 5A). These cells were then grafted subcutaneously into the nude athymic mice (n = 4) and tumor size was estimated weekly using a caliper. As shown in Figure 5B, tumors resulting from cells expressing reduced levels of OTUB1 (Figure 5C), exhibited significant delay in tumor growth, with tumor sizes of only 27% as compared to controls. Accordingly, tumors bearing OTUB1 shRNA showed lower levels of the proliferation marker Ki-67 (Figure 5C). Finally, we assessed the occurrence of metastases in mice grafted orthotopically in the prostate. As shown in Figure 5D and 5E, PC3-shOTUB1 cells exhibited reduced orthotopic tumor growth and reduced incidence of lymph node metastases. Interestingly, PC3-shOTUB1 grafted mice only exhibited lymph node metastatic foci while PC3-shControl grafted mice also showed metastatic foci in liver and kidney.

## Discussion

In this study we have demonstrated that OTUB1 protein levels are elevated in prostate cancer and contribute to the induction of cell invasion through a mechanism that involves the activation of RhoA. The analysis of xenograft models also demonstrates that OTUB1 can modulate tumor growth and metastatic development *in vivo*.

OTUB1 belongs to the OTU domain containing family of cysteine-dependent proteases (OTUDs). OTUB1 is able to cleave the isopeptide bond within polyubiquitin chains, with a preference for Lys48 linked ubiquitins [22], while other members of the family have specificity for other types of ubiquitin linkages [22,29-31]. Given that most of the studies exploring OTUDs activity utilize short poly-ubiquitin polymers unattached to target proteins, it is unclear whether they reflect the functions of these proteins *in vivo*. Indeed, recent studies have shown that OTUB1 can negatively regulate Ubc13 mediated Lys63 linked ubiquitination of RNF168 in a manner that is independent of its protease activity and dependent on a direct interaction through the N-terminal domain of the protein [15,32]. Similar results were obtained in U2OS cells where overexpression of both WT and the OTUB1-C91S inactive mutant led to an increased expression of p53 through interference with UBCH5/MDM2 mediated ubiquitination [33]. Therefore, through its interactions with E2 ubiquitin ligases, OTUB1 can modulate biological processes independently of its proteolytic activity. This is particularly interesting when considering our observations that in prostate cancer cells, OTUB1 mediates the androgen inhibition of p53 through a mechanism that requires an intact OTUB1 catalytic motif and seems to be independent from MDM2 concentration changes (Figure 3B). Moreover, in LNCaP-FGC cells cultured in the absence of androgens, OTUB1 depletion is unable to alter p53 levels (Figures 3B and 4E). In contrast, OTUB1 is required for androgen signaling leading to the inhibition of p53 expression. Overall, our findings suggest that OTUB1 regulation of p53 in prostate cancer cells is indirect and the result of altered androgen signaling. Therefore, the mechanisms whereby OTUB1 regulate p53 seem to change in tissue and stimuli specific manner.

We found that in prostate cancer cells, OTUB1 has the capacity to modulate cell invasion of both AR+ and AR- cells in a manner that seems to be dependent on the presence of an intact active site. While overexpression of WT OTUB1 leads to increased cell invasion, the expression of the OTUB1-C91S variant mimics the effects of OTUB1 siRNA knockdown, leading to reduced cell invasion (Figure 1). Similar effects were observed when RhoA activity was measured, suggesting that in addition to the non-canonical effects of OTUB1 previously described, its proteolytic activity is important for some of its biological actions, in line with what have been shown for the regulation of ER alpha levels by this protein [34].

In previous studies we have shown that androgens can regulate cell invasion [3,11]. Here we demonstrate that the ability of androgens to induce the activation of RhoA is modulated by OTUB1 and leads to an increased cell invasion capacity as well as downregulation of p53 (Figure 3B). The antagonistic relationship between p53 status and Rho activity is well known, as it has been demonstrated that either loss of p53 or expression of p53 mutants promotes the activation of RhoA and the induction of cell invasion [28]. These findings seem to place RhoA downstream of p53 actions. On the other hand, there is also evidence for alternative mechanisms where signaling molecules involved in the regulation of cell motility can influence p53 function. For example, it has recently been shown that JMY which function as an actin nucleation factor in the cytosol to promote cell motility has also important activities as a transcriptional co-activator of p53. The enhanced translocation of JMY into the nucleus upon DNA damage negatively regulates motility while enhancing p53 function [35,36]. In androgen receptor expressing LNCaP-FGC prostate cancer cells, androgens´ negative regulation of p53 levels also relies on the regulation of cell motility pathways depending of RhoA activation. While siRNA mediated downregulation of p53 in androgen depleted cells cause minor effects on RhoA activation, the knockdown of RhoA is sufficient to enhance p53 expression in androgen treated cells.

We have previously reported the ability of OTUB1 to proteolytically remove ubiquitin moieties from (presumably Lys48-linked) polyubiquitinated RhoA *in vitro*, suggesting that OTUB1 actions on RhoA activity may be the consequence of direct actions leading to reduced proteasomal degradation and stabilization of RhoA [27]. This interpretation is now challenged by our findings that OTUB1 downregulation has little effects on RhoA cellular content, while proteasome inhibition leads to reduced rather than increased activation of RhoA in response to androgen treatment (Additional file 2: Figure S2). This finding suggests that the proteasome may be involved in the inactivation of RhoA inhibitors in LNCaP-FGC cells, which may be directly or indirectly targeted by OTUB1. Given the myriad of regulatory proteins controlling RhoA activity, it is difficult to perform a comprehensive analysis of the effects of OTUB1 in these pathways. In this context it is relevant to mention that a recent study have demonstrated that androgens activate RhoA in muscle cells by promoting its membrane translocation subsequent to the polyamination of RhoA. Therefore, the modulation of polyamine metabolisms by OTUB1 needs to be considered among the possible mechanisms explaining its effects on RhoA [37]. A number of protein-protein interaction screenings has only identified a handful of OTUB1 interacting proteins, with not known function as RhoA regulators, questioning the assumption that OTUB1 has domains for the specific recognition of enzymatically targeted proteins in addition to those that bind Lys48 linked polyubiquitin [22]. Our own attempts to investigate an interaction between RhoA and OTUB1 in LNCaP-FGC cells by multiple means have failed to demonstrate a direct association. Therefore, additional studies are needed to identify the mechanisms used by OTUB1 to regulate RhoA function.

The biological functions of OTUB1 are poorly understood, in part because of the lack of sufficient insight into which proteins or biological processes are modulated by this DUB. Here, we provide the first demonstration that OTUB1 protein levels are altered in human prostate cancer (Figure 2) and that downregulation of OTUB1 expression limits tumorigenesis *in vivo* (Figure 5)*.* Our findings on the role of OTUB1 in the regulation of RhoA and p53 activity suggest that these are relevant pathways to explain the effects of OTUB1 in tumor growth. Ample amount of evidences has linked prostate cancer progression to loss of p53 function [38]. Moreover, a significant overlap exists between the genomic changes associated with different stages of prostate cancer progression with those induced by oncogenic RhoA mediated transformation (Additional file 3: Figure S3), suggesting that this is an important pathway for prostate tumor progression in humans. Interestingly, we also demonstrated that downregulation of OTUB1 levels leads to effects on AKT1 and eNOS phosphorylation that antagonize those induced by DHT. Future studies should address whether these changes are relevant for OTUB1 effects on tumorigenesis and the mechanisms whereby OTUB1 regulates these proteins.

## Conclusions

In summary, we demonstrate that OTUB1-mediated activation of RhoA promotes cell invasion of prostate cancer cells. OTUB1 also promotes tumorigenesis *in vivo* in good agreement with the overexpression observed in prostate cancer tumors. Thus, our results would support the investigation of drugs targeting the catalytic activity of OTUB1 as potential therapy for advance prostate cancer.

## Methods

### Cell lines, materials and plasmids

LNCaP-FGC and PC-3 PCa cell lines were purchased from ATCC (Rockville, MD) in 2010 and cultured as described previously [3]. Dihydrotestosterone (DHT) was obtained from Amersham (Braunschweig, Germany). Bortezomib and MG132 were obtained from LC Laboratories (Woburn, MA, USA). Sea plaque agarose for soft agar experiments was purchased from Cambrex (Rockland, ME, USA). The plasmids bearing the HA-tagged OTUB1 allele and the C91S mutant have been described previously [27]. The functional screening was performed using siRNA pools purchased from Santa Cruz Biotechnology (Santa Cruz, CA, USA). For RhoA Knockdown shRNA pools from Santa Cruz Biotechnology (Santa Cruz, CA, USA) were used. OTUB1 individual siRNAs were purchased from Qiagen (Hilden, Germany) Transfections were performed using the Neon transfection system (Invitrogen, Carlsbad, CA, USA) according to the manufacturer's instructions. Stable PC3 cell lines were generated by transfecting the SureSilencing shRNA plasmid for human OTUB1 (Qiagen, Hilden, Germany) encoding the Neomycin resistant gene and the pGL4.17-[luc2/puro] (Promega, Madison, WI, USA), PC3-shOTUB1, or the same plasmid containing a scramble sequence which does not match any human gene, PC3-shControl. Expression vectors for WT-RhoA, Q63L-RhoA and DN-RhoA were described previously [39].

### RNA extraction, cDNA synthesis and real-time–PCR

These procedures were carried out as earlier described [3]. All measurements were performed in triplicate for each sample and normalized to the internal control gene, *β-*actin. Four independent experiments were performed. The primers are listed in Additional file 4: Table S1.

### Western blotting

The western blotting procedure was carried out as described earlier [10]. Mouse anti-human OTUB1 antibody was obtained from Cell Signaling (Fremont, CA, USA). Mouse monoclonal rose against p53 (D0-7) and p21 (F-5), rabbit polyclonal anti-RhoA (119) and human β-actin (sc-130301) antibodies were from Santa Cruz Biotechnology (Santa Cruz, CA, USA) and mouse monoclonal anti-RhoA (ARH03-A) was from Cytoskeleton (Denver, CO, USA).

### Phospho-antibody arrays

Profiling of phosphorylation changes of 46 different signaling proteins in response to DHT treatment or OTUB1, siRNA mediated knockdown was performed using the Proteome Profiler antibody array (ARY003) from R&D systems (Minneapolis, MA, USA), using the manufacturer’s instructions. Signal intensities were quantified by densitometry using ImageJ software [40].

### Cell proliferation assay

LNCaP-FGC cells were placed in phenol red-free RPMI 1640 containing 5% DCC-FBS and cultured for 24 h before stimulation with 10 nM DHT or a vehicle control. PC3 cells were cultured in RPMI 1640 containing 10% fetal bovine serum. Proliferation was measured using the xCelligence system (Roche Diagnostics, Indianapolis, IN, USA), following manufacturer’s recommendations. Cells were continuously monitored during 96 h and doubling times calculated based on the impedance values at time 24 h and 96 h. At least three independent experiments were performed.

### Matrigel invasion assay

This procedure was carried out as previously described [3]. Four independent experiments were performed. In each set of experiments every group was measured in triplicate.

### Growth in soft agar

This assay was carried out as previously described [11]. Each plate was seeded with 10^5^ cells. Every 3 days, 0.5 ml of fresh media was added to the cells. After 14 days, the top layer of the culture was stained with 0.2% iodonitrotetrazolium chloride, and colonies larger than 0.1mm in diameter were counted.

### Small GTPase activity assay

RhoA activity was analyzed using the Rho Activation Assay Biochem Kit from Cytoskeleton (Denver, CO, USA) following manufacturer instructions. The assay is based on the measurement of GTP-bound RhoA isolated using Rhotekin bound beads. Likewise, Rac and Cdc42 activity was measured using commercially available kits (G-LISA Cdc42 and Rac Activation assay Biochem Kit, Cytoskeleton).

### Immunohistochemistry

Tissue micro arrays (TMAs) including formalin-fixed, paraffin-embedded samples from men diagnosed with prostate cancer between 1975 and 1995 after transurethral resection of the prostate were analyzed, and have been previously described in detail [41]. TMAs for analysis were selected to represent tumors of different grades; Gleason score (GS) 5 (n = 4), GS 6 (n = 17), GS 7 (n = 18), GS 8 (n = 12), GS 9 (n = 15), and GS 10 (n = 4). TMA sections, 4 μm thick, were used for IHC essentially as reported [41], with OTUB1 (1:75, Cell Signaling) as primary antibody. The epithelial cell staining was scored as weak, moderate, or strong in each TMA core, and the general (median) score for non-malignant and malignant cores was reported per patient. The predominant cellular localization of the staining was recorded. The research ethic committee in Umeå, Sweden, approved this study.

### Quantitative proteomic profile

LNCaP-FGC cells were metabolically labeled following the stable isotope labeling by amino acids in cell culture (SILAC) methodology [23]. Briefly, cell were cultured for at least 10 generations in RPMI medium (Biowest) supplemented with 10% dialyzed FBS (Sigma) and 28 and 48 mg/L of stable isotopic variants of arginine and lysine respectively (Cambridge isotope laboratories, Inc) as only source for these amino acids. Cells cultured with Arg10 (^13^C_6_; ^15^N_4_) and Lys8 (^13^C_6_; ^15^N_2_) were denominated “heavy”, “medium” when the amino acids were Arg6 (^13^C_6_;) and Lys4 (4,4,5,5-D4) and “light” with Arg0 and Lys0. All amino acids were purchased from Cambridge isotope laboratories, Inc. “Light” cells were transfected with siRNA control while “heavy” and “medium” cells were transfected with different siRNA targeting OTUB1. Whole cell extracts were purified and mixed in a 1:1:1 ratio and resolved by SDS-PAGE. After trypsin in-gel digestion, peptides were analyzed by LC-MS/MS using an orbytrap mass spectrometer. The obtained mass spectrometric raw data were analyzed in the MaxQuant environment [42] with the integrated Andromeda searching engine and false discovery rate cut-off for peptide identification of 0.1 [43]. The effects of OTUB1 depletion were calculated as the result of dividing the normalized intensity of each “heavy” (siOTUB1_1) and “medium” (siOTUB1_2) protein by the intensity of the correspondent “light” (siControl) protein.

### Mice xenograft models

Prostate Cancer PC3-shOTUB1 and PC3-shControl cells were grafted in athymic nude mice either subcutaneously or orthotopically into the anterior prostate as described [44]. In brief, the abdomens of 8-week-old anesthesized athymic nude mice were surgically opened under sterile conditions. PC3-shOTUB1 and PC3-shControl cells (5 × 10^6^) suspended in 50μl of Matrigel were injected into one lobe of anterior prostate by 25-gauge needle and the abdomens were closed by silk sutures (4 mice per group). In another set of mice (PC3-shControl, n = 4; PC3-shOTUB1, n = 5) cells were implanted with matrigel subcutaneously. Growth of tumors was monitored weekly using a caliper and tumor volume was calculated as described [45]. Mice harboring tumors in the prostate were killed 8 weeks later and the size of orthotopic prostate tumors was measured. The number of metastatic foci was counted. Tissues were fixed and embedded in paraffin for further analyses. Immunohistochemical detection of OTUB1 and Ki-67 was performed as described [46] using antibodies from Cell signaling (OTUB1, 1:75) and Abgent (Ki-67, 1:100). All the experiments were conducted in compliance with the policies and regulations of Tianjin Medical University Institutional Animal Care and Use Committee (Tianjin, China). Ethical permit SYXK (Tianjin) 2009-0001.

## Additional files

Additional file 1: Figure S1. OTUB1 depletion inhibits 22Rv1 cells invasion. Measurements of matrigel invasion of 22Rv1 cells transfected with either siRNAs targeting OTUB1 or control siRNA. ^#^ indicates a significant (*p*<0.05) change between cells transfected with siOTUB1 versus control transfected cells. Additional file 2: Figure S2. Chemical inhibition of the 26S proteasome results in reduced amount of active RhoA. LNCaP cells were starved in 5% charcoal-striped serum for 24h and then treated with 10 nM DHT for 8h with or without proteasome inhibitors bortezomib or MG132 at the indicated concentrations. GTP-bound RhoA was pulldown from whole cell extracts using Rhotekin-RBD beads. Western blot analysis of pulldowns and whole cell extracts was performed to determine the expression of the indicated proteins. Additional file 3: Figure S3. Significant overlap between genes regulated in prostate cancer and genes regulated by RhoA. (A) Gene Set Enrichment Analysis (GSEA, Broad Institute, Cambridge, MA, USA) of the genes with elevated (left panel) or reduced (right panel) expression levels in localize prostate tumors versus benign prostate tissue [47] were compared with genes with increased or decreased expression due to the overexpression of a constitutively active form of RhoA (RhoA-Q63L) [48]. (B) Genes with elevated (left panel) or reduced (right panel) expression levels in prostate cancer-derived metastasis [47] versus localize prostate tumors were compare as in (A) with the same set of RhoA modulated genes. False Discovery Rate “*q*” value is shown. Additional file 4: Table S1. Primers used in this study for RT-PCR.

## Abbreviations

PCa: Prostate cancer
OTUB1: Otubain 1
DUBs: Deubiquitinating enzymes
CRPC: Castration resistant prostate cancer
AR: Androgen receptor
DHT: Dihydrotestosterone
GS: Gleason score
TMA: Tissue microarray
SILAC: Stable isotope labeling by amino acids in cell culture
