# Erratum: OTUB1 de-ubiquitinating enzyme promotes prostate cancer cell invasion *in vitro* and tumorigenesis *in vivo*

**DOI:** 10.1186/s12943-015-0341-1

**Published:** 2015-04-18

**Authors:** Diego Iglesias-Gato, Yin-Choy Chuan, Ning Jiang, Charlotte Svensson, Jing Bao, Zhiqun Shang, Indranil Paul, Lars Egevad, Benedikt M Kessler, Pernilla Wikström, Yuanjie Niu, Amilcar Flores-Morales

**Affiliations:** The Novo Nordisk Foundation Center for Protein Research, Faculty of Health Sciences, University of Copenhagen, 2200 Copenhagen, Denmark; Tianjin Institute of Urology, Tianjin Medical University, 300211 Tianjin, China; Department of Surgical Science Karolinska Institutet, Section of Urology, 17176 Stockholm, Sweden; Nuffield Department of Clinical Medicine, Target Discovery Institute, University of Oxford, OX37BN Oxford, UK; Department of Pathology, Umeå University, 90185 Umeå, Sweden

## Erratum

After publication of this study [1], we found that we unintentionally failed to provide the complete list of all coauthors. This error has now been corrected and the full list of authors contributing to this work is included. Authors’ contributions and Competing interests sections have been modified accordingly.
