# Supplementary material for: OTUB1 de-ubiquitinating enzyme promotes prostate cancer cell invasion in vitro and tumorigenesis in vivo
Source: Mol Cancer. 2015 Jan 27;14(1):8. doi: 10.1186/s12943-014-0280-2 (PMC4320819; doi:10.1186/s12943-014-0280-2)
Supplement: Additional file 4: Table S1. — Primers used in this study for RT-PCR. [file 12943_2014_280_MOESM4_ESM.doc]

Table S1

Primers used in this study for RT-PCR.

| OTUB1-F | GTC TGC CAA GAG CAA GGA AG |
| --- | --- |
| OTUB1-R | GCT TCT CCA CCT GCT CAA TC |
| OTUD2-F | CTT GAA AAA GTA GGG ATG GGC |
| OTUD2-R | ATC AAT GCA TCT TAA TGA CAA T |
| OTUD3-F | GCC AGC CCT AGT GAA GAA AA |
| OTUD3-R | CCA AGA GGC CAA AGA GTC AG |
| OTUD4-F | ATC TTA GCC GGA CAC CTT CA |
| OTUD4-R | TGG GAA AGC CTG TTC ATC TC |
| OTUD5-F | ATC CTA CCT GCA GTG GTT GC |
| OTUD5-R | TAA AAC AGT GCC TGG GGA AG |
| OTUD7B-F | AGG ATC ACA TGA CCC TGG AC |
| OTUD7B-R | CCA CCA CTC CCC TCA CTA AA |
| OTUD7C-F | GCG TTC AGG ACA CAG ACTT G |
| OTUD7C-R | TTC ATC ATT CCA GTT CCG AGT ATC |
| TRABID-F | GAA GAT TTG CCC CCA ACA GTC C |
| TRABID-R | AGC TTG CTC CAG GCT GAC TAG C |
| b-actin F | CTGGCTGCTGACCGAGG |
| b-actin-R | GAAGGTCTCAAACATGATCTGGGT |
